# Increasing walking in patients with intermittent claudication: Protocol for a randomised controlled trial

**DOI:** 10.1186/1471-2261-10-49

**Published:** 2010-10-07

**Authors:** Maggie A Cunningham, Vivien Swanson, Ronan E O'Carroll, Richard J Holdsworth

**Affiliations:** 1 Department of Psychology, University of Stirling, Stirling, UK; 2 Department of Surgery, Stirling Royal Infirmary, Livilands Gate, Stirling, UK

## Abstract

**Background:**

People with intermittent claudication are at increased risk of death from heart attack and stroke compared to matched controls. Surgery for intermittent claudication is for symptom management and does not reduce the risk of cardiovascular morbidity and mortality. Increasing physical activity can reduce claudication symptoms and may improve cardiovascular health. This paper presents the pilot study protocol for a randomised controlled trial to test whether a brief psychological intervention leads to increased physical activity, improvement in quality of life, and a reduction in the demand for surgery, for patients with intermittent claudication.

**Methods/Design:**

We aim to recruit 60 patients newly diagnosed with intermittent claudication, who will be randomised into two groups. The control group will receive usual care, and the treatment group will receive usual care and a brief 2-session psychological intervention to modify illness and walking beliefs and develop a walking action plan. The primary outcome will be walking, measured by pedometer. Secondary outcomes will include quality of life and uptake of surgery for symptom management. Participants will be followed up after (a) 4 months, (b) 1 year and (c) 2 years.

**Discussion:**

This study will assess the acceptability and efficacy of a brief psychological intervention to increase walking in patients with intermittent claudication, both in terms of the initiation, and maintenance of behaviour change. This is a pilot study, and the results will inform the design of a larger multi-centre trial.

**Trial Registration:**

Current Controlled Trials ISRCTN28051878

## Background

Patients with Peripheral Arterial Disease (PAD) have been described as 'a high risk, but neglected, disease population' [1]. The disease is common in older adults, has serious health implications, but is not as intensively treated as other cardiovascular diseases [2]. PAD is caused by atherosclerosis in the leg arteries, which leads to a reduction in blood supply to the lower limbs. The most common symptom of PAD is intermittent claudication (IC), leg pain when walking. The prevalence of PAD increases with age, Fowkes et al [3] found that 4.5% of 55 to 74 years olds had IC in the Edinburgh Artery Study, with prevalence increasing to over 20% of over 75 year olds [4].

Atherosclerotic build-up on the walls of arteries is not confined to one part of the arterial system, therefore a diagnosis of IC is an indicator of atherosclerosis in other parts of the body. Indeed, patients diagnosed with IC are at increased risk of cardiovascular co-morbidity and mortality. Caro et al [5] analysed a large database of patients with PAD and found that 33% of patients with PAD died, mainly from cardiovascular events, within 5 years of diagnosis of IC. A diagnosis of IC can therefore be seen as an important early warning sign of future cardiovascular problems, and should trigger measures to reduce the risk of death and vascular co-morbidity [6].

Many of the risk factors for atherosclerosis are behavioural, and include smoking, lack of physical activity, high cholesterol levels, high blood pressure, diabetes and obesity. Treatment for patients diagnosed with IC includes risk factor modification and surgery. Walking benefits patients with PAD as it reduces blood pressure, improves the lipid profile of the blood, and encourages development of a collateral blood supply [7]. In a Cochrane review of exercise for intermittent claudication, Leng et al [8] found significant improvements in walking distance following exercise therapy, the most effective exercise regimens involving walking to near-maximal pain, three times a week. Leng called for further research to determine the degree of supervision required in an exercise programme, and how long changes in exercise could be expected to last following participation in an exercise programme.

At present in the UK, supervised exercise programmes for patients with IC are relatively uncommon, therefore exercise for claudication is usually promoted by advice alone. Stewart & Lamont [9] indicated a number of reasons why advice alone may not be appropriate for patients with IC, including patients' loss of confidence/self-efficacy in their ability to walk, their embarrassment at having to stop and rest, and their concerns that pain may be an indication that walking is harmful to them. The aim of this RCT is to test a brief psychological intervention designed to improve walking in patients with IC.

In our recent qualitative study of factors associated with walking in patients with IC, we found that two belief systems influenced walking - (1) beliefs about the illness, and (2) beliefs about walking [10]. We found that patients with claudication tended to have a poor understanding of the causes of their illness, the health consequences of their illness, or the benefits of walking either to their general health or to reduce claudication symptoms.

While some previous studies using brief psychological interventions to increase physical activity in populations at high risk of developing cardiovascular disease have had disappointing results e.g. [11-13], the present study differs because (i) the intervention is designed to address the barriers to walking particular to patients with IC, whereas other studies have included heterogeneous groups of patients facing a wide range of unknown barriers to physical activity; (ii) the patients included in this study have sought treatment for their disease and are therefore presumably more motivated to adhere to treatment than patients being offered unsolicited interventions to increase physical activity; (iii) this study will objectively directly measure behaviour change, in the form of mean daily steps as measured by pedometer, rather than relying on self-report measures of physical activity, or objective measures of fitness as a proximal measure of behaviour change; (iv) this study will measure all outcomes at three time points, 4 months, 1 year and 2 years from baseline, thereby distinguishing between initiation and maintenance of behaviour change - previous studies have not tended to measure all outcomes at all time points and therefore cannot distinguish between the initiation and maintenance of behaviour change.

### Framework

Our intervention was designed using the MRC Framework for the development and evaluation of complex interventions to improve healthcare [14]. We identified existing evidence for the benefit of walking therapy to patients with claudication, and have conducted a qualitative study to identify key psychological constructs associated with walking in claudicants. Our theoretical framework is based around Leventhal et al's [15] Common-sense model of self-regulation of health and illness (CSM), a dynamic model which considers both the role of illness representations and treatment representations in an individual's coping actions. The CSM suggests that patients' illness representations are based on beliefs about the causes of the illness, how long the illness will last, whether the illness is curable, and the perceived consequences of the illness. Treatment representations can be characterised using the same framework as illness representations - behavioural actions have an identity (e.g. the name of the action), time lines (e.g. the length of time the behaviour will take to achieve the goal, and when and for how long the behaviour has to be carried out), consequences (e.g. outcome expectations about the consequences of the behaviour; judgement about the risk of performing the action), causes (e.g. the mechanism by which the coping action will work) and control (e.g. whether the action will cure or control symptoms or the underlying disease; the individual's beliefs about their ability to carry out the behaviour). The model proposes that the content of these representations determines the cognitive and behavioural actions which the individual will take to cope with the illness. We have identified evidence-based behaviour-change techniques successfully used in other interventions to modify the psychological constructs highlighted in the CSM [16].

This trial is a pilot to test whether a brief psychological intervention to modify these constructs can lead to an increase in walking in IC patients. In the pilot study we will test recruitment and retention rates, preliminary efficacy of the intervention at both short and long-term follow-up, acceptability of the intervention, and acceptability of pedometers as a way of measuring walking behaviour in this patient group. The results from this pilot trial will provide an effect size to inform the power and sample size calculations for a larger, multi-centre trial.

### Trial objectives

To examine whether:

• a brief psychological intervention can improve walking and quality of life for patients with intermittent claudication;

• a brief psychological intervention can reduce the demand for surgery for symptom management;

• improvement in walking is mediated by changes in illness and walking beliefs;

• participation in the intervention improves surgical outcomes e.g. walking behaviour and quality of life, for participants who have surgery.

## Methods/Design

### Recruitment

Participants will be recruited from the Intermittent Claudication Outpatient Clinic in NHS Forth Valley. This is a nurse-led clinic which diagnoses people with IC. People are referred from GPs based in Forth Valley. At diagnosis, the Vascular Assessment Nurse will invite consecutive patients to take part in the trial. In order to check representativeness of the participants who agree to take part in the trial, the Vascular Assessment Nurse will ask for verbal consent to retain all patients' age, gender and Ankle Brachial Pressure Index information, whether they agree to participate in the trial or not.

Currently 200 patients are diagnosed each year in the Forth Valley Intermittent Claudication clinic. A sample size calculation carried out using GPower3 [17], indicates that to carry out a (2) group by (2) time mixed ANOVA to detect a small effect size (0.2) with an alpha of 0.05, and a power of 80%, the trial would require 26 participants in each condition. A small effect size of 0.2 was selected in the sample size calculation based on a previous study of an exercise program for physical activity in patients with IC which measured walking outcomes using pedometers [18]. The sample size calculation was based on the conservative initial (2) time point (baseline to 4 month) mixed ANOVA to increase the likelihood that the study was adequately powered to identify an initiation of walking behaviour change. A (4) time point mixed ANOVA sample size calculation required fewer participants in the study, and therefore the larger, initial (2) time point, more conservative sample size calculation was used. The sample size calculation was based on changes in the primary outcome measure. As this is a pilot study, a further aim is to determine the effect sizes achieved in the secondary outcome measures, to help inform the sample size calculation for a larger, more definitive study. Based on a previous study with patients from this population recruited at outpatient clinic, we estimate that 25% of people may decline to participate in the study. Likely attrition rate from the study for this population is unknown, therefore we have estimated a 10% attrition rate during the trial. We therefore aim to invite 80 patients to participate in the trial, of whom we expect 60 to agree to participate, and a further 6 to drop-out during the course of the trial. Based on typical numbers presenting at the clinic who are diagnosed with claudication, recruitment is estimated to take place at the rate of 2 participants/week.

### Inclusion Criteria

Patients presenting to the Forth Valley Vascular Outpatient Clinic, newly diagnosed with IC in one or both legs will be recruited into the study. A diagnosis of lower limb arterial disease causing intermittent claudication will be established by a vascular assessment nurse or consultant surgeon. This will be based on a combination of investigations including post-exercise ABPI of below 0.9 [6], and duplex ultrasound, magnetic resonance angiography (MRA) or computerised tomography angiography to provide an anatomic view of the arteries.

### Exclusion Criteria

We will only exclude people who are unable to give informed consent (e.g. due to dementia) or for whom it may be medically unadvisable to increase their daily walking, including patients with heart failure, cancer, patients who cannot walk unaided or patients with a history of orthopaedic surgery which affects walking. Patients with an ABPI of less than 0.35 will also be excluded as they are likely to be offered emergency surgery.

### Design

**Figure 1:**
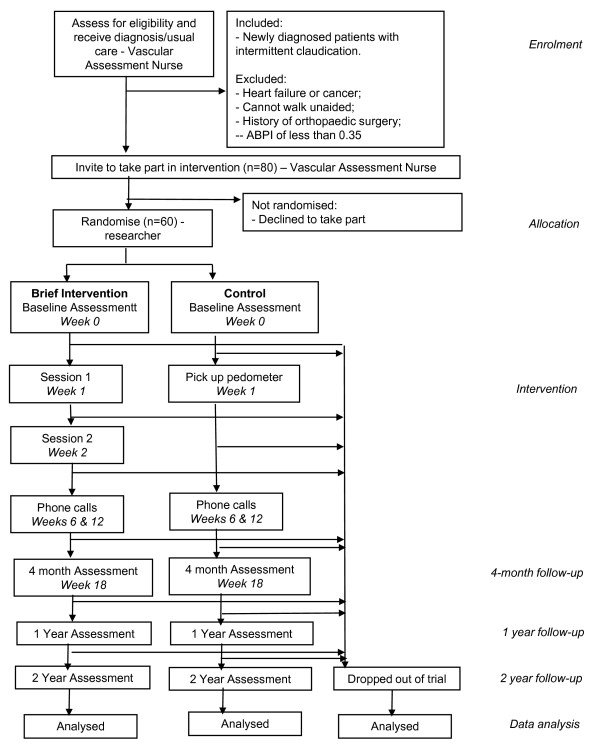
**CONSORT flowchart of trial design**.

This is a pilot randomised controlled trial with patients newly diagnosed with IC randomly allocated to one of two groups. Allocation to group will be carried out by the researcher, who will use a computer generated random number table for allocation (http://www.randomizer.org/). The computer generated random number table was created at the start of the study by a research colleague, who is not involved in participant recruitment or allocation. The researcher will assign consecutive participant numbers to patients as their names are sent to her by the Vascular Assessment Nurse, each participant number will then correspond to a treatment group as established in the computer generated random number table. The researcher will therefore assign participants to groups before she has made any contact, and before she has received any information about them other than their name and phone number. Participants allocated to the control condition will receive usual care. Participants allocated to the intervention group will receive usual care and a brief, 2-session psychological intervention. The Vascular Assessment Nurse who provides usual care at diagnosis, and who recruits participants, will be blind to participant allocation to groups. Other clinical staff in contact with participants during their care, e.g. Vascular Technologists and Vascular Surgeons will be blind to participant allocation to groups. A CONSORT flowchart of the trial design is shown in Figure 1.

### Setting

This is a single-centre trial based in NHS Forth Valley, Scotland.

### Ethical Approval

Ethical approval has been obtained from Fife and Forth Valley Research Ethics Committee (REC ref. no. 08/S0501/6).

### Intervention

Participants in the intervention group will receive 9 visits from the researcher in their own home, including 4 visits to complete questionnaires, 2 one hour intervention sessions, and 3 other visits to drop off or pick up pedometers.

#### Session 1

The researcher will elicit any maladaptive beliefs the participant has about their illness, or about walking. Information will be provided about the consequences of the illness, and about the behaviour-health link in order to modify illness and walking beliefs. Motivational interviewing techniques will be used to deliver the intervention, asking open questions, prompting the participant to make self-motivating statements and evaluations of their own walking behaviour and aiming to move participants to a point where they have expressed motivation or intention to change their walking behaviour. A copy of the schedule used to guide the discussion in Session1 is provided in Additional File 1.

#### Session 2

The researcher will work with the participant to draw up an individualised walking action plan, based on the recommendation of walking to near maximal pain three times a week [8]. Participants will be encouraged to think about how they can adapt their daily routines to incorporate a half hour of continuous walking, and will also be encouraged to think of possible barriers which may prevent them from following their action plan, and strategies to cope with these barriers. Sniehotta et al [19] found that a combination of action planning and coping planning led to much greater improvement in physical exercise than action planning alone. Action plans will then be typed up and laminated, and posted out to participants. A copy of the template for an action plan is provided in Additional File 2.

### Control

Participants in the control group will receive 8 visits from the researcher, in their own home, including 4 visits to complete questionnaires and a further 4 visits to either drop off or pick up the pedometer. This is only 1 less visit from the researcher than participants in the intervention group. The researcher will engage control group participants in non-walking related conversation in an attempt to control for the potentially confounding effects of social contact.

### Usual Care

Participants in the intervention group and control condition will receive usual care while participating in the study. All participants will be diagnosed as having intermittent claudication by the Vascular Assessment Nurse, who provides all patients with an information sheet about Peripheral Arterial Disease and advises patients to 'stop smoking and start walking'. All participants then attend an outpatient clinic to meet with a Vascular Surgeon, who will discuss appropriate treatment with the patient. Appropriate treatment depends on the age and health of the patient, the size and location of the narrowing of the artery, and on the patient's wishes. If the patient chooses surgical intervention they will then be put on a waiting list for surgery or angioplasty. Approximately 56% of patients diagnosed with IC in NHS Forth Valley proceed to receive angioplasty or surgery, usually within 6 months of diagnosis. If the patient chooses conservative treatment, they will be invited to attend a further outpatient clinic in 3 months time, and then discharged if their claudication symptoms remain stable. Patients who receive angioplasty are followed up by the surgeon for 1 year and then discharged; patients who receive surgery are followed up by the surgeon for 5 years.

At the time of 4 month follow-up in the RCT, all patients will have attended the outpatient clinic to meet the Vascular Surgeon, and a treatment decision will have been made. However, patients will be on a waiting list and will not have received treatment.

### Telephone Calls

The researcher will call participants in both groups, 6 and 12 weeks after recruitment into the study. The purpose of the call will be to:

• Intervention group - discuss progress against the action plan with participants in the intervention group, playing particular attention to barriers to walking, and encouraging participants to use their coping plans, and come up with alternative strategies to overcome barriers. Changes to a participant's action plan or coping plan may be agreed with a participant in the phone call.

• Both groups - ask participants if they have heard from the hospital and what treatment, if any, has been agreed upon.

• Both groups - ask participants to answer 5 standard questions about general health, satisfaction with health, quality of life [20], pain rating [21], and pain free walking distance [22].

### Outcome Measures

Measures will be taken for participants in both arms of the study, at baseline, 4 months, 1 and 2 year follow-ups. All questionnaires will be administered by the researcher.

### Primary Outcome - walking

Previous studies of walking in patients with intermittent claudication have tended to measure maximal walking distance on the treadmill. While this measure gives an accurate idea of how far a patient can walk before they experience claudication pain, and before they have to stop, it does not give any idea of how much walking the patient does in their day to day life, and therefore lacks ecological validity. For this reason, we have decided to measure day-to-day walking using pedometers. Participants will be asked to wear a pedometer for one week at each time point, and their mean daily steps will be calculated by averaging the six days with the highest number of steps.

Patients will be shown how to use the pedometer at each time point, and will be encouraged to telephone the researcher if they have any problems with the pedometer.

### Other Measures

Self-reported walking ability will be measured with the question 'How far can you walk, aided or unaided, under normal circumstances before the onset of pain?' to which there are 6 possible responses - '0 yards', 'up to 100 yards', 'up to 250 yards', 'up to half a mile', 'up to 1 mile', 'more than a mile'. The question is adapted from Mondillo et al's [22] Claudication Self-Assessment with the distances being re-formatted from metres to yards to reflect the fact that older patients are more likely to be confident measuring distance in imperial units. In order to measure frequency of walking to the onset of pain, participants are asked 'In general, how frequently do you walk that distance?' with 6 possible responses - 'never', 'once or twice a month', 'once a week', '2-3 times a week', '4-5 times a week', and 'everyday'.

A self-report measure of walking will also be completed, as a comparison for walking as measured by pedometer. The International Physical Activity Questionnaire (IPAQ) [23] is a self-report measure of physical activity with 4 items measuring time spent in vigorous and moderate activity, time spent walking, and time spent sitting.

Quality of life will be measured using the Intermittent Claudication Questionnaire [21] a disease specific quality of life measure.

Illness beliefs will be measured using the Brief Illness Perception Questionnaire [24], adapted to reflect Peripheral Arterial Disease as the illness in question.

There is no standard measure of treatment representations, however walking personal control (walking confidence) questions will be tailored to people with intermittent claudication based on methods described in Francis et al [25]; and treatment consequences (walking) questions will be tailored to people with intermittent claudication based on methods described in Renner & Schwarzer [26].

We will also document all surgery received since the last visit.

### Analysis

Data will be analysed using an intention to treat protocol in a repeated measures mixed design (2 groups*2 time points for short-term outcome, and 2 groups*4 time points for long-term outcome). Our aim is to determine the effect size achieved by this pilot to inform the sample size calculation for a larger, multi-centre study. Between-group differences in uptake of surgery will be measured using a chi-square test.

### Evaluation

The effectiveness of the intervention will be determined through analysis of the outcome variables listed above. Analysis of the primary outcome variable will determine whether the brief psychological intervention group have increased walking in comparison with the control group. The psychological measures will be analysed to determine whether improvement in walking is mediated by changes in illness and walking beliefs.

On completion of the study, participants in the intervention group will be asked additional semi-structured questions about their experience of the intervention including what they thought of their action plan, whether they followed their action plan, whether the barriers they predicted prevented them from following their action plan, whether they used their coping plan, whether they intend to follow their action plan in the future, and whether they thought it was worthwhile to take part in the study.

## Discussion

A brief psychological intervention which increases walking in claudicants may (a) reduce the demand for surgery for patients with intermittent claudication; (b) reduce costs for the NHS; (c) be accessible and acceptable for patients and lead to long term behaviour change; (d) encourage patient self-management of their chronic condition; and (e) increase patient quality of life.

This trial is novel in that previous interventions to increase walking in claudicants have either relied on the provision of advice, which does not overcome barriers to walking for claudicants; or supervised exercise programmes, which are not commonly available and which may not be an acceptable means of intervention for many claudicants for a variety of psychological and environmental reasons. This is the first time a psychological intervention based on the CSM has been carried out to increase walking in patients with intermittent claudication. The intervention is patient-centred, can be tailored to the specific needs of each individual, and has directly measured behaviour change (walking) as the primary outcome. A health economic evaluation would be central to any subsequent large-scale trial.

A clear theoretical framework underpins the intervention, and the content of the intervention is clearly specified and based on thoroughly tested and evidenced health psychology techniques. The intervention is capable of being delivered by trained non-specialist health workers in an NHS setting.

## Competing interests

The authors declare that they have no competing interests.

## Authors' contributions

MC conceived of the study, designed the study and drafted the manuscript. VS, REO'C and RJH participated in the design of the study. All authors read and approved the final manuscript.

## Pre-publication history

The pre-publication history for this paper can be accessed here:

http://www.biomedcentral.com/1471-2261/10/49/prepub

## Supplementary Material

Additional file 1 **Questions for Session One**. Schedule to guide Session One, using questions based on motivational interviewing techniques to discuss the participant's beliefs about their illness, provide information on the illness, provide information on the benefits of walking, and discuss the participant's motivation to change their walking behaviour. Click here for file

Additional file 2 **Template for Action and Coping Plan**. Template for an action and coping plan to be completed with the participant by the researcher, in the participant's own words. The plan can include three specific actions which the participant will undertake to increase their walking. Prompts for discussing possible barriers to action are included in the template. The template is adapted from the Improving Health: Changing Behaviour - NHS Health Trainer Handbook [27]. Click here for file
