# Supplementary material for: Increasing walking in patients with intermittent claudication: Protocol for a randomised controlled trial
Source: BMC Cardiovasc Disord. 2010 Oct 7;10:49. doi: 10.1186/1471-2261-10-49 (PMC2958933; doi:10.1186/1471-2261-10-49)
Supplement: Additional file 1 — Questions for Session One. Schedule to guide Session One, using questions based on motivational interviewing techniques to discuss the participant's beliefs about their illness, provide information on the illness, provide information on the benefits of walking, and discuss the participant's motivation to change their walking behaviour. [file 1471-2261-10-49-S1.DOC]

**Additional File 1 Questions for Session One**

**What is your understanding of intermittent claudication?**

(I have some information about intermittent claudication, would you like to hear it?)

**Looking at the risk factors, what do you think you can do about your circulation problems?**

**What are you thinking about your current walking behaviour at this point?**

**How would you like to be in 6 months time?**

**What would be the good things about doing more walking?**

**Rate on a scale of 0 to 10 (with 10 being the highest) how interested you are in making a change to your walking?**

(Why did you not choose a higher number?)

**Rate, again on a scale of 0 to 10, how confident you are that you can make the change?**

(What would it take to get you to a higher number?)

**What personal strengths do you have that will help you succeed?**

**Who could offer you support in making this change?**
