# Supplementary material for: Increasing walking in patients with intermittent claudication: Protocol for a randomised controlled trial
Source: BMC Cardiovasc Disord. 2010 Oct 7;10:49. doi: 10.1186/1471-2261-10-49 (PMC2958933; doi:10.1186/1471-2261-10-49)
Supplement: Additional file 2 — Template for Action and Coping Plan. Template for an action and coping plan to be completed with the participant by the researcher, in the participant's own words. The plan can include three specific actions which the participant will undertake to increase their walking. Prompts for discussing possible barriers to action are included in the template. The template is adapted from the Improving Health: Changing Behaviour - NHS Health Trainer Handbook [27]. [file 1471-2261-10-49-S2.DOC]

**Additional File 2 Template for Action and Coping Plan**

**The changes I want to make are:**

…………………………………………………………………………………………..

…………………………………………………………………………………………..

…………………………………………………………………………………………..

**The most important reasons why I want to make these changes are:**

…………………………………………………………………………………………..

…………………………………………………………………………………………..

…………………………………………………………………………………………..

**My general goal is:** ……………………………………………………………………………………………………………………………………………………………………………………………………………………………………………………………………………………………..

**My first specific action**

WHAT am I going to do?

WHERE am I going to do it?

WHEN am I going to do it?

WITH WHOM am I going to do it?

3 possible barriers which might hinder me from doing this activity?

1.

2.

3.

How am I going to overcome these barriers? (substitution of means, increased effort etc)

**My second specific action**

WHAT am I going to do?

WHERE am I going to do it?

WHEN am I going to do it?

WITH WHOM am I going to do it?

3 possible barriers which might hinder me from doing this activity?

1.

2.

3.

How am I going to overcome these barriers? (substitution of means, increased effort etc)

**My third specific action**

WHAT am I going to do?

WHERE am I going to do it?

WHEN am I going to do it?

WITH WHOM am I going to do it?

3 possible barriers which might hinder me from doing this activity?

1.

2.

3.

How am I going to overcome these barriers? (substitution of means, increased effort etc)

**I will know my plan is working if:**

…………………………………………………………………………………………..

…………………………………………………………………………………………..

…………………………………………………………………………………………..

…………………………………………………………………………………………..

Notes:

Re barriers, ask:

- Is there anything about the things around you or the places you are in that makes it difficult to do this behaviour? What can you do to change this?
- Are there any people I spend time with who make it difficult to do this behaviour? What can I do to change this?
- Is there anything I am thinking or feeling that makes it difficult to do this behaviour? How can I overcome these things?
